# COVID-19 and fertility services in the United Kingdom: a biphasic qualitative study

**DOI:** 10.1530/RAF-20-0055

**Published:** 2021-03-01

**Authors:** B Karavadra, A Stockl, A H Balen, E P Morris

**Affiliations:** 1 Norfolk & Norwich University Hospital, Obstetrics & Gynaecology, Colney Lane, Norwich, Norfolk, UK; 2 University of East Anglia, Norwich Medical School, Norwich Research Park, Norwich, Norfolk, UK; 3 The Leeds Teaching Hospital, Obstetrics & Gynaecology, Great George Street, Leeds, UK; 4 Norfolk & Norwich University Hospital, Obstetrics & Gynaecology, Colney Lane, Norwich, Norfolk, UK

**Keywords:** infertility, experiences, qualitative research, COVID-19 and fertility

## Abstract

Recently, fertility services have started resuming since COVID-19 was declared a pandemic, but there remains significant uncertainty in the way this care will be delivered in the United Kingdom. The objective of our study was to explore the impact of COVID-19 on individuals using fertility services in the United Kingdom. The study was conducted in two phases between May 2020 and July 2020: an online questionnaire involving 1212 participants and subsequent individual semi-structured telephone interviews with 15 participants. Through thematic analysis, we learned from the questionnaire findings that 74% of individuals identified as White British, 21% as Black and Minority Ethnic (BAME) and 2.6% as male. Ninety-six per cent of individuals from the questionnaire explained that COVID-19 had a ‘negative impact’ on their fertility treatment, namely ‘delay in care’. Eighty-two per cent of participants discussed concerns about the ‘uncertainty’ they felt about fertility services; these included the ‘unknown impact of COVID-19 on pregnancy outcomes’, the ‘unknown impact on general gynaecology services’ and the ‘unknown impact of COVID-19 on fertility success’. Through semi-structured telephone interviews with 15 participants, we learned about the ‘cultural pressures’ individuals from BAME backgrounds faced in relation to care. Participants were mindful about the ‘pressures on the service’ when reopening, and therefore ‘advancing maternal age’, ‘socio-economic background’ and ‘previous unsuccessful fertility treatment’ were the main factors individuals considered important when ‘prioritising’ fertility care. Our findings can be used by fertility service providers to appreciate the patient perspective when considering the reopening of fertility services nationally and internationally.

**Lay summary:**

The impact of COVID-19 on patients seeking or undergoing fertility treatment is not entirely known. Many patients have had their treatment postponed during the pandemic. As fertility services begin to recommence, it is important to understand how the pandemic has affected this group of patients. In addition, it is vital to appreciate and understand the patient’s voice in order to ensure services take into account the patients’ concerns as they begin to offer certain fertility treatments. Our study was conducted in two phases and involved an online questionnaire and individual interviews with people. We found that people were worried about services restarting and how care would be prioritised. People also discussed some of the perceived barriers to seeking fertility healthcare. Our findings highlight the importance of understanding the patient’s voice when recommencing fertility services.

## Introduction

The COVID-19 pandemic has had an unprecedented impact on the world. Fertility services in the United Kingdom were temporarily suspended when the pandemic first started. In addition, routine gynaecological workload was also stopped throughout the country. Consequently, patients who were about to undergo fertility treatment, or indeed, midway through it, had to have it stopped.

Previous public health crises including H1N1 swine flu, HIV/AIDS and the West African Ebola (Brolin Ribacke 2016) epidemic have all had a significant impact in the way healthcare is delivered, although they have not affected the provision of IVF in the United Kingdom. The COVID-19 pandemic started in Wuhan, China, in Nov/Dec 2019, with the first reported case in the United Kingdom in late January 2020 and the first deaths in the United Kingdom in March 2020. It was soon noted that measures had to be taken to create clinical capacity within acute hospitals for the pandemic and ensure that the clinical and nursing staff were appropriately prepared, trained and if necessary redeployed. The British Fertility Society (BFS) and the Association of Reproductive and Clinical Scientists (ARCS) issued guidance concerning the care of fertility patients during the pandemic, first in the form of a statement on 16 March 2020 and then a guideline on 18 March 2020. On 16 March, the ARCS and BFS made the recommendation to temporarily suspend elective assisted conception treatment (British Fertility Society). A national lockdown involving social distancing measures and restrictions in travel was announced on 23 March by the Prime Minister.

The regulatory authority of IVF clinics in the United Kingdom (the Human Fertilisation and Embryology Society, HFEA) published General Direction 0014, instructing clinics to complete active treatments by 15 April 2020. Concurrently communication from the Chief Executive and Chief Operating Officer of the NHS directed all NHS providers to, amongst other measures, prepare to ‘postpone all non-urgent elective operations from 15 April at the latest, for a period of at least 3 months’.

On Friday, 1 May, the Secretary of State for Health and Social Care announced that fertility clinics would be allowed to open from May 11. The HFEA allowed clinics to apply from May 11 to resume licenced activities. A small number of clinics were able to recommence treatments shortly thereafter, with appropriate measures to reduce risk with distancing and reduced footfall through clinics. From the 15 April 2020, all non-urgent elective operations were postponed for at least 3 months (British Fertility Society).

The resumption of services has been variable and slow, as some clinical spaces were used for other purposes and many staff were redeployed to work in other parts of the health service (not only nursing and medical but also embryology and administrative). Even by August 2020 some clinics were yet to reopen.

The impact of the shutdown on service users is not exactly known and therefore this study was designed to explore this impact. The objective of the study was to explore participants’ experiences of fertility care during COVID-19 and any perceived barriers to care and to gain insight into the way participants felt about fertility care upon resumption of services. The study was conducted in two phases: phase one being an online questionnaire and phase two being individual semi-structured telephone interviews.

## Materials and methods

### Study design

This study involved an exploratory qualitative approach through two phases. Phase one involved the use of a context-setting online questionnaire (the questionnaire is presented in Supplementary information 1, see section on supplementary materials given at the end of this article) and phase two with individual semi-structured interviews (Supplementary information 2) over the telephone. The study design, questionnaire development and semi-structured interview guide were developed with the support of three clinicians, a sociologist, four patients who were waiting to undergo fertility treatment and the Chief Executive of Endometriosis UK. Consensus on the items for inclusion was reached as a team through discussion.

Through the use of various online platforms and radio broadcast messages, the questionnaire was distributed nationally. The questionnaire was initially piloted on ten participants, and feedback was obtained to make amendments as necessary (for instance, the structure of sentences and the use of terminology). In addition to demographic data, the questionnaire content included participants’ experiences of fertility care in the United Kingdom, their perceptions of COVID-19 and its impact on their fertility care and perceived barriers to fertility care. Consent was obtained online by participants clicking on the questionnaire link and ticking the respective disclosure.

The findings from the questionnaire study were used to inform the second phase of the study that involved individual semi-structured interviews with participants selected through purposive sampling. Interviews are a common method of obtaining qualitative data (Whiting 2008). In addition to clinician, patient and public involvement for the design of the semi-structured interview guide, the findings from the questionnaire also guided its development. Consensus on items for inclusion was once again reached as a group. The interviews itself were conducted by BK and designed to further explore the findings from phase one in much greater depth. Each recorded telephone interview lasted between 30 and 108 min. A semi-structured interview guide was used (Appendix 1). Topics identified from the online questionnaire findings for further exploration in the interviews included perceived barriers to fertility care, participants’ view on the extension of embryo storage and their experiences of fertility care.

### Study population

Table 1 shows the demographic details for the questionnaire respondents and semi-structured interview participants.

**Table 1:** Demographic details for the questionnaire respondents (*n* = 1212) and semi-structured interviews (*n *= 15). Data are presented as *n* (%).

Demographic	Questionnaire	Semi-structured interview
Age		
18–24	5 (0.4%)	1 (6.6%)
25–34	889 (73.3%)	9 (60%)
35–44	316 (26%)	5 (33.3%)
45–54	2 (0.16%)	0
55–64	0	0
64+	0	0
Sex		
Male	32 (2.64%)	5 (33.3%)
Female	1174 (96.8%)	9 (60%)
Other	6 (0.49%)	1 (6.66%)
Geographical location		
East of England	182 (15%)	3 (20%)
East Midlands	101 (8.3%)	3 (20%)
London	133 (10.9%)	1 (6.66%)
North East	121 (9.98%)	1 (6.66%)
North West	98 (8.08%)	1 (6.66%)
Northern Ireland	11 (0.90%)	0
Scotland	97 (8.00%)	1 (6.66%)
South East	147 (12.1%)	2 (13.3%)
South West	133 (10.9%)	0
Wales	21 (1.73%)	0
West Midlands	76 (6.27%)	2 (13.3%)
Yorkshire and the Humber	92 (7.59%)	1 (6.66%)
Ethnicity		
White/White British	898 (74%)	8 (53.3%)
Black/Black British	52 (4.29%)	2 (13.3%)
Asian/Asian British	181 (14.9%)	4 (26.6%)
Mixed race	19 (1.56%)	1 (6.66%)
Rather not say	1 (0.08%)	0
Another ethnicity	61 (5.61%)	0
Highest level of education		
Less than secondary school	12 (0.99%)	3 (20%)
Secondary school	321 (26.4%)	3 (20%)
Some university, but no degree	111 (9.15%)	1 (6.66%)
Foundation degree	31 (2.55%)	0
Bachelor’s degree	698 (57.5%)	7 (46.6%)
Postgraduate degree	39 (3.21%)	1(6.66%)
Relationship status		
Heterosexual	1108 (91.4%)	12 (80%)
Same sex	66 (5.44%)	2 (13.3%)
Single person	29 (2.39%)	1 (6.66%)
Other	9 (0.74%)	0

### Statistical analysis

The findings from the questionnaire were analysed quantitatively through percentages, and the open-ended questions from the questionnaire through qualitative thematic analysis (Alhojailan 2012, Javadi & Zarea 2016). The data were analysed separately by two researchers and through the use of a qualitative research software NVivo 1.0 (QSR International; www.qsrinternational.com/nvivo-product). NVivo is a software that is used commonly in qualitative research to organise large amounts of qualitative data. Thematic analysis involved a number of stages including (1) familiarising with the data set (reading through the responses, making notes on an excel file), (2) generating initial codes (ascribing labels to the open-ended responses), (3) generating and refining themes (grouping the different codes together under common themes) and (4) relating the themes to the context of the study (the findings were related to the clinical context by the researchers) (Nowell *et al.* 2017).

The findings from the telephone interviews were transcribed verbatim and analysed through thematic analysis as discussed above by two researchers independently and then collaborated together. Data saturation was reached with 15 participants and this was defined as no new themes being generated from the analysis. Data saturation was defined as no new themes being generated from the data collection method (Saunders *et al.* 2018). Whilst 15 interviews may appear to be a small number, in qualitative research, this number generated a significant amount of data. Importantly, the inter-coder reliability between both researchers was 96.2%. If there were any discrepancies, then this was discussed with a third researcher.

### Ethical approval

The study was approved by the Faculty of Medicine and Health Sciences Research Ethics Committee at the University of East Anglia. Reference: 2019/20-120.

## Results

The combined findings from both aspects of the study are represented as nine themes (Table 2). The questionnaire results are discussed as percentages and these findings are further qualified by quotes from the semi-structured interviews (these are represented as brackets with the respective interview number).

**Table 2:** Main themes generated from the study.

Theme	
Impact of COVID-19
Getting pregnant during COVID-19
The changing nature of fertility services
The 10-year storage limit
Uncertainty in treatments
Uncertainty in service resumption
Being diagnosed with COVID-19
Concerns about male fertility treatment
Perceived barriers to accessing care

One thousand four hundred and one participants clicked on the online questionnaire link, but 1212 participants replied and completed the questionnaire. Fifteen participants were recruited online to the second phase of the study involving individual semi-structured interviews.

Based on the questionnaire findings, 98% of participants used a fertility clinic in the United Kingdom within the past 12 months. Table 3 shows the most recent fertility experience and Table 4 shows the most recent type of fertility treatment that participants underwent. During the COVID-19 pandemic, the majority (88% of participants) were under National Health Service (NHS) fertility care and the remainder under private care.

**Table 3:** Fertility experience participants (*n *= 1212) had prior to fertility services temporarily stopping.

Fertility experience	*n*
With a male partner	1136
With a female partner	38
No partner	29
Supporting another individual	5
Supporting another couple	4
Other	0

**Table 4:** Most recent fertility treatment participants (total *n* = 1212) underwent or had planned to undergo prior to COVID-19 lockdown.

Fertility treatment	*n*
Fertility preservation	442
*In vitro* fertilisation	721
Donor insemination	31
Intrauterine insemination	3
Unsure	15

The nine themes generated from the questionnaire findings are presented below. These include ‘impact of COVID-19’, ‘getting pregnant during COVID-19’, ‘the changing nature of fertility services’, ‘the 10-year storage limit’, ‘uncertainty in treatments’, ‘uncertainty in service resumption’, ‘being diagnosed with COVID-19’, ‘concerns about male fertility treatment’ and ‘perceived barriers to accessing care’. As the semi-structured interviews were guided by the questionnaire findings, verbatim quotations from the interviews were used to exemplify the main themes further.

### Impact of COVID-19 on patients

Ninety-two per cent of participants from the questionnaire stated that their fertility care had been negatively impacted due to COVID-19. Reasons for this included complete cessation in treatment, not being kept up to date with when and how services may resume and uncertainty in how the virus may impact pregnancy outcomes.

### Getting pregnant during COVID-19

Seventy-eight per cent of participants in the questionnaire were ‘worried about getting pregnant during COVID-19’. Reasons for this included concerns as to whether contracting COVID-19 would result in a miscarriage, whether patients needed to self-isolate if they were pregnant due to IVF and if COVID-19 would result in ‘congenital problems’ if pregnant.

It just feels so scary to get pregnant during Covid. On one hand, I want to get pregnant and have a baby, but on the other, I am scared at the thought of catching Covid and it potentially affecting the pregnancy. It makes me want to just wait until things settle. Delaying everything has really been on my mind. With Covid, is it bad that I am more worried about my potential pregnancy than myself? (Interview 14, age 21, female, White British)

There is so much information about Covid and how it affects people. But, there just doesn’t seem to be much about the way Covid can affect pregnancy and if there are any known future complications from it. My partner and I just really worry. (Interview 13, age 41, female, mixed race)

### The changing nature of services upon resumption

Eighty-eight per cent of participants from the questionnaire perceived fertility care changing in the way services will be delivered once they resume again. Participants had concerns about how their care would resume once services recommenced, if clinics will be taking any extra precautions in the form of personal protective equipment and what measures services will have in place to ensure participants do not contract COVID-19. Participants also discussed concerns as to whether certain fertility services will ever resume in the way they were previously.

I know that things will have to change due to Covid. I get that. But that doesn’t stop me from worrying whether I will be able to have my IVF. I just don’t know how things will ever get back to normal. It just makes everything feel so hopeless. (Interview 6, age 28, female, White British)

I have read so many news articles saying that people just don’t have the right sort of PPE. This worries me a lot. I am so keen to get on with my treatment, but I am also worried that I will be putting myself at risk of catching Covid from hospitals. (Interview 15, age 40, other gender, White British)

### Ten-year storage limit

Participants were asked to comment on their thoughts about the 10-year storage limit on frozen embryos, eggs and sperm. In the questionnaire findings, over 80% of participants felt the additional 2-year storage limit was a positive decision, but some participants discussed their anxieties.Eight participants from the semi-structured interviews explained that an extension implied to them that future fertility care will be delayed upon resumption. One participant felt that the extension was implying that fertility services cannot cope with the patient demand and needed ‘more time to help people’. Participants discussed the following:

I mean, it’s great that people are allowed to have their eggs for example stored for two years longer, but the fact that the government have done this makes me anxious that there will be a delay in restarting my treatment as the extra two years may be needed. (Interview 8, age 34, female, White British)

The two-year extension is all very well, but does that mean that I will have to wait longer before I am seen again to start my treatment? (Interview 6, age 28, female, White British)

I just think that the government have introduced this new policy as they can’t cope with the patient demand. More and more people want fertility care, but how can they fit everyone in? This is their way of pretending that they are helping patients, but I don’t think they are really. (Interview 12, age 42, female, Black British)

### Uncertainties on what treatments can be offered

Ninety-one per cent of participants from the questionnaire were concerned about what fertility treatments will be offered and to whom and when services will recommence. Participants in the interviews expanded on this:

I am worried what sort of fertility treatments my centre will be able to restart again. Even when they do restart, I wonder if they be prioritising it to certain people. There was already stigma in my community from being childless already and this has made it worse. (Interview 1, age 31, female, Asian British Indian)

Surely things will take a while to return to normal and even with the different treatments, they can’t possibly get everyone in on time. There will be some criteria I bet on who gets what first. (Interview 4, age 36, male, White British)

From both the questionnaire and interview findings, participants expressed concern about the potential variation in treatments amongst fertility centres. They were also worried about how fertility services would be prioritised amongst patients. Participants discussed factors that they felt were important when deciding how services should be prioritised. These included ‘advancing maternal age’, ‘socio-economic background’ and ‘previous unsuccessful fertility treatment’.

### Uncertainties in the way services will resume again

Participants were worried about how centres would begin the process of restarting their treatment. During semi-structured interviews, participants discussed their anxieties about routine gynaecology care:

I am due to have a keyhole test on my fallopian tubes to check if they are working properly, but at the same time, they said I can have some endometriosis treated. I wonder how long this keyhole surgery will now take because of COVID. (Interview 3, age 29, female, Asian British Indian)

I have been waiting for a laparoscopy for such a long time. I guess this will never happen now. (Interview 8, age 34, female, White British)

### Concerns about being diagnosed with COVID-19 and its impact on fertility treatment

Participants through both phases of the study expressed concern about being diagnosed with COVID-19. They were worried about the impact the virus would have on any developing pregnancy as well as long-term implications on a live baby. Concerns were also expressed as to whether a miscarriage was more likely or not in a COVID-positive patient.

### Concerns about male fertility treatments

From both aspects of the study, participants discussed concerns about the way male fertility services may be delivered. Male participants in the interviews explained that they perceived official communication from services provides as ‘female orientated’ and that there was less focus on the needs of male patients.

It is great that all the official organisations are trying to keep everyone updated about fertility treatment etc, but I have found that there doesn’t seem to be much focus on men who are undergoing fertility treatment specifically. I have had cancer before and so there just doesn’t seem to be much information on how my fertility care might be affect or how I might be feeling. (Interview 10, age 39, male, White British)

### Perceived barriers to care

Participants from a Black and Minority Ethnic (BAME) background discussed the ‘cultural pressures’ they faced during COVID-19 and the implications on their wider social circumstances. Through semi-structured interviews, they expressed concerns about being ‘disadvantaged’ from a health equalityt perspective and felt ‘more at risk’ from pregnancy-related complications. Many had read about health outcomes in those individuals from a BAME background who were diagnosed with COVID-19. Participants in the semi-structured interviews expressed concerns as to whether ethnicity in general influenced fertility outcomes.

As routine gynaecology work was also suspended, participants discussed this as a barrier to fertility treatment. Some participants were waiting for benign gynaecological surgery prior to fertility care. Other barriers included ‘not being kept updated about service resumption’ by some fertility centres, perceived ‘lack of interest in fertility care by the government’ and the influence of the media. To expand on this, participants felt that the media focussed primarily on how COVID-19-related care was delivered as opposed to fertility care. As a result of the uncertainty in the way services may be delivered, some participants considered ‘postponing’ their care until the pandemic was ‘more stable’.

## Discussion

### Summary of principal findings

Through 1212 questionnaires and 15 semi-structured interviews, our findings not only provide insight into the negative impact COVID-19 has had on participants’ fertility care but also highlight the professional support they have or, in the majority of cases, have not received during the closure of services. During the semi-structured interviews, we gained in-depth understanding and awareness of the barriers and concerns participants perceived to their care as services began to slowly resume particularly for those patients who were identified as male. In addition, some participants discussed how they were impacted by the cessation of routine benign gynaecological surgery. In addition, we demonstrate the absolute need for clinicians and other fertility care providers to be mindful of the changing needs of this group of patients as they may require further emotional, medical and psychological support during their treatment(s). Our findings highlight the importance of providing information that is tailored to specific concerns related to fertility and COVID-19 to patients undergoing fertility care.

During COVID-19, fertility clinics responded in different ways by alerting their patients through websites, social media, and in some cases, through personal telephone calls to keep them updated on their respective services. Furthermore, some clinical spaces were utilised for other medical functions and staff were redeployed to other clinical and non-clinical duties. Patients found this information useful as they also felt informed. Whilst a position statement released by the British Fertility Society provided insight into the resumption of fertility services in the United Kingdom, uncertainty in its exact delivery still remains. In addition, should a second wave of COVID-19 occur, there do not appear to be contingency plans *in situ* for the way fertility services will be delivered. The latest WHO report on COVID-19 also does not make reference to the resumption of fertility services. As routine gynaecological surgery was also postponed during the pandemic, the government and secondary care services will need to explore how they will be able to manage this workload once services resume.

### Strengths and weakness of the study

Numerous reports were released through the British Fertility Society on how fertility services can be reintroduced. However, to our knowledge, there are no other published studies in the United Kingdom involving semi-structured interviews that explored the impact of COVID-19 on individuals who are undergoing or about to undergo fertility treatment. Our study provides significant insight into the way participants perceived COVID-19 to have influenced their care and the support they received during the pandemic from their respective fertility centre. Participants also discussed the perceived barriers to care and their concerns about resuming fertility services. The findings from our study highlight the importance of the patient’s voice when considering the resumption of fertility services.

Due to COVID-19 and the social restrictions imposed by the government, recruitment to the study was via online media. As a result, our findings may have captured those individuals who are social media active and therefore may under-represent the findings from those who are not. Whilst attempts were made to ensure a fair representation from individuals from a variety of demographics, the overall majority of participants were from a White British background and therefore this may have influenced the findings.

The number of replies from individuals who identified as male were also low, and as such the views of this group were under-represented. The findings from this UK study may not necessarily represent the views from those individuals throughout the world. Finally, the study was conducted during May 2020 to July 2020 and therefore the findings are representative of a time where our knowledge on COVID-19 was limited. Since this time, knowledge in this area in relation to pregnancy in particular has advanced.

### Findings in relation to other studies

Our findings echo similar messages from other studies. Vaughan *et al*. (2020) found that during COVID-19 infertility was the main stressor that participants were concerned about. Boivin *et al*. (2020) provide a very interesting insight into the coping mechanisms used by fertility patients during the pandemic. Both studies demonstrate why our study is important for those providing fertility care throughout the world. Our study is unique in that it involved interviews with participants, and we were able to explore further insightful information in detail, particularly relating to barriers in accessing fertility care and how participants’ perceived care should be prioritised upon resumption of services.

### Future research

Our study provides the basis for further qualitative work. Undoubtedly, it may take some time before services resume back to the ‘pre-COVID-19’ era and therefore, services may need to be prioritised amongst patients. How this prioritisation is done is a question for stakeholders, clinicians and academics to decide with the engagement of patient and public involvement.

## Conclusion

The impact of COVID-19 has been far reaching for many individuals. For those undergoing fertility care, it has been a particularly challenging and unpredictable time. This study has truly enabled participants’ voices to be heard. The findings from this study can be used by fertility service providers to appreciate the patient perspective when considering the reopening of fertility services nationally and internationally and be mindful of patient concerns.

## Supplementary Material

Supplementary Information 1

Supplementary Information 2

## Declaration of interest

E P M has received a grant from Gedeon Richter as the UK Chief Principle Investigator for the PREMIUM study. E P M has also received consultancy fees from Chugai Pharma. E P M has been an adviser for Pfizer in 2018 and Gedeon Richter in 2019. No other conflict of interest for any of the other authors.

## Data availability

The data underlying this article will be shared on reasonable request to the corresponding author.

## Author contribution statement

B K conceived the study, obtained ethical approval and was involved in the design, execution, analysis, manuscript drafting and critical discussion. A S was involved in the analysis, manuscript drafting and critical discussion. A B and E P M were involved in the design, manuscript drafting and critical discussion. All authors approved the final version of the manuscript.
