# Supplementary material for: COVID-19 and fertility services in the United Kingdom: a biphasic qualitative study
Source: Reprod Fertil. 2021 Mar 1;2(1):27–34. doi: 10.1530/RAF-20-0055 (PMC8812411; doi:10.1530/RAF-20-0055)
Supplement: Supplementary Information 1 [file supplementary_table_1.pdf]

Supplementary Information 1

Questionnaire

An online questionnaire study to explore the impact of COVID-19 on fertility services in the United Kindom.

Patient information

Thank you for clicking on the survey Monkey link. Below, you will find some information about the study. Please have a read and tick the box at the end to confirm you are happy to proceed to the consent page. Following the consent page, the questionnaire will begin. It should not take more than 20-30 minutes to complete.

Why is this study being done?
Covid-19 has become a pandemic. We are aware that this has resulted in so many changes in the way the world is functioning. In particular, social distancing has affected the way in which patients seek help; particularly those who are undergoing fertility treatment. We want to gain more information into your experiences as either a pregnant woman recently undergone fertility treatment or who is currently undergoing fertility treatment.

What do I have to do?
You will be asked to complete an online questionnaire via Survey Monkey. This questionnaire is available on this very link you have clicked on once you have agreed to participate in the study.

What are the risks of taking part?
There are no identifiable risks to you in taking part. At the end of the questionnaire, you will be provided with some online links and a reminder of who to contact should you have any concerns about your care.

Are there any benefits to me taking part?

You will be providing valuable insight into your fertility care experiences during this unprecedented time. We hope that you will also gain insight into what you think about the current Covid-19 pandemic and how it will affect your care.

How will my results be used?
The findings will provide insight into any potential changes to care that can be made on a local or national level. A lay summary of the findings will also be available on the Royal College of Obstetricians and Gynaecologists website as well as presented at a conference and in a research journal.

How will confidentiality be maintained?
All the data you provide will be stored in a password protected computer and in a password protected file. None of the data you provide will be identifiable to you personally. The data will be stored for a total of 10 years, in line with the University of East Anglia policy. Survey monkey will not generate any information that will identify you personally and confidentiality will remain at all times.

Who has developed this study and has it been approved?
This work is being led by researchers at the University of East Anglia. The study has been approved by the Research Ethics Committee at the University of East Anglia

Further information and contact details
Should you have any further questions, please contact Dr Babu Karavadra. He is current a trainee in Obstetrics and Gynaecology and is leading this piece of research. His email address is b.karavadra@uea.ac.uk

Resources
Please see below some online resources you may find useful:

The Royal College of Obstetricians and Gynaecologists: https://www.rcog.org.uk/en/patients/
The Royal College of Midwifery: https://www.rcm.org.uk/about-us/

Consent form
Please read the following points and tick the box at the end of the statement to confirm you are happy with the consent form and you do not have any questions or concerns about the study.

1. I wish to take part in this online study
2. I am aware that the findings from my questionnaire will be kept confidential under the current GDPR regulations
3. I have read the participant information section above and do not have any questions.
4. I have had the chance to ask any questions in relation to the study and do not have any other queries.
5. All the information that I am providing is entirely voluntary and I am aware that I can withdraw my consent for the data use prior to submitting my results at the end of the questionnaire
6. I am aware that once I have submitted my data at the end of the questionnaire, I cannot withdraw my data

I am happy with the above consent points and do not have any questions about the study ☐

Demographics
Below are some questions in relation to your demographics.

1. **How old are you?**
2. **Where in the UK are you from? (Select)**
3. **What is your ethnicity, if any**
4. **If you are employed, what is your current profession?**
5. **Which of the following best describes your gender?**

General

6. **Have you ever used a fertility clinic in the United Kingdom?**

- Yes

- No

7. **What was your most recent fertility experience based as?**
- With a male partner
- With a female partner
- No partner
- Supporting another individual
- Supporting another couple
- Other

8. **If you have, when was the last time you used it?**
- Within 1 year
- More than 1 year ago
- More than 2 years ago
- More than 3 years ago

9. **What type of fertility treatment did you undergo?**
- Fertility preservation
- Invitro fertilisation
- Donor insemination
- Intrauterine insemination
- Unsure
- Other

10. **When you were considering your fertility choices, where did you find information from? (white space)**

11. **Which one of the following best describes where you obtained fertility care most recently from:**
    - NHS
    - Private
    - Other

<u>**Covid-19 and fertility care**</u>

12. **Has Covid-19 had a negative impact on your IVF care?**
    Yes
    No

13. **If yes, please can you provide more detail as to why? (white space)**

14. **Where did you find information about fertility choices during Covid-19? (white space)**

15. **Do you have any concerns about getting pregnant during Covid-19?**
    Yes
    No

16. **If yes, please can you describe them in the box below (white space)**

17. **Do you think the Covid-19 pandemic will change the way in which fertility care is delivered in the UK?**

18. **If yes to question 16, please can you describe more in the box below**

19. **Some patients have had a 10-year storage limit on frozen eggs, sperm, and embryo's. The recent government advice has meant there has been a 2-year extension on this. If this applies to you, have you been made aware?**
    Yes/No/Not applicable

20. **In relation to question 18, what are your thoughts on the 2-year extension?**

21. **Once fertility services resume, individuals will be able to contact their centre for more information. In your opinion, do you think care should be prioritised in anyway?**
    Yes/No/Unsure

22. **Please can you explain your answer to question 20 in the box below**
23. **During your most recent fertility care experience, have you experienced any barriers to having your care?**
    Yes/No/Not applicable

24. **If yes to question 22, please can you describe more in the box below?**

25. **Please can you tell us any other thoughts or concerns you have about fertility care in the UK in relation to Covid-19 (white space)**
