# Supplementary material for: COVID-19 and fertility services in the United Kingdom: a biphasic qualitative study
Source: Reprod Fertil. 2021 Mar 1;2(1):27–34. doi: 10.1530/RAF-20-0055 (PMC8812411; doi:10.1530/RAF-20-0055)
Supplement: Supplementary Information 2 [file supplementary_table_2.pdf]

Supplementary Information 2

Semi-structured interview guide: A study to explore participants experiences of fertility services during the COVID-19 lockdown; a biphasic study.

Introduction

- Thank you for agreeing to take part in this telephone interview. We are interviewing you to explore the way COVID-19 may have affected you and your fertility care. There are a number of areas we wanted to ask questions about. If at any point you want to stop, then please let me know. It is also important to remember that your participation in the study today will not affect your care in anyway.
- Thank you for returning the signed consent form. Do you have any questions or queries?
- Participating in the study is entirely voluntary. Everything we discuss will be typed word by word, however, no information will identify you personally.

Establishing rapport throughout the interview with prompts and signposting as necessary.

Topics of discussion

- Questions about demographic data. Age/Ethnicity/Geographical location/Level of education/Sexual orientation (as already mentioned in the information leaflet)/Type of fertility care under consideration/in-situ.
- Personal journey so far with infertility/subfertility.
- Experiences of fertility care in the United Kingdom: including support provided by their fertility centre during the pandemic.
- Experiences of fertility care in the United Kingdom during COVID-19.
- Thoughts about any concerns about fertility services resuming.
- Thoughts about any perceived barriers to care.
- Any other thoughts

Conclusion

- Thank you for partaking in this telephone interview. Your experiences are extremely valuable. Do you have any questions or concerns before we finish the interview today? Thank you.
